# Immunoglobulin G passive transfer from mothers to infants: total IgG, IgG subclasses and specific antipneumococcal IgG in 6-week Malawian infants exposed or unexposed to HIV

**DOI:** 10.1186/s12879-022-07335-0

**Published:** 2022-04-05

**Authors:** Silvia Baroncelli, Clementina M. Galluzzo, Stefano Orlando, Robert Mphwere, Thom Kavalo, Richard Luhanga, Roberta Amici, Marco Floridia, Mauro Andreotti, Fausto Ciccacci, Maria Cristina Marazzi, Marina Giuliano

**Affiliations:** 1 National Center for Global Health, Istituto Superiore di Sanità, Viale Regina Elena, 299, 00161 Rome, Italy; 2 Department of Biomedicine and Prevention, University of Rome Tor Vergata, Rome, Italy; 3 DREAM Program, Community of S. Egidio, P.O. Box 30355, Blantyre, Malawi; 4 Saint Camillus International, University of Health Sciences, Rome, Italy; 5 Department of Human Sciences, LUMSA University, Rome, Italy

**Keywords:** Placental transfer, IgG, IgG subclasses, HIV-exposed infants, Anti-PCP IgG

## Abstract

**Background:**

The impaired transplacental passage of IgG from mothers living with HIV to their infants could be one of the causes of the high vulnerability to infections of HIV-exposed uninfected (HEU) infants, but controversial results have been obtained in different settings. The aim of this study was to assess in 6-week old HEU and HIV-unexposed, uninfected (HUU) Malawian infants the total IgG levels, the subclasses profile and the concentrations of global anti-pneumococcal capsular polysaccharide (anti-PCP) IgG and IgG2.

**Methods:**

Dried blood spots were collected from 80 infants (40 HEU, 40 HUU) and antibodies concentrations determined by nephelometric method (total IgG and subclasses), or using ELISA (anti-PCP total IgG and IgG2). Results are expressed as median levels with IQR, while the proportions of each subclass out of the total IgG are used to describe the subclasses profile*.*

**Results:**

At 6 weeks HEU infants had higher median levels of total IgG and IgG1 and a significantly lower level of IgG2 [0.376 (0.344–0.523) g/l vs 0.485 (0.374–0.781) g/l, p = 0.037] compared to the HUU counterparts. The IgG subclasses distribution confirmed the underrepresentation of IgG2 (IgG2 represented 5.82% of total IgG in HEU and 8.87% in HUU). The anti-PCP IgG and IgG2 levels were significantly lower in HEU infants [8.9 (5.4–15.1) mg/l vs 16.2 (9.61–25.8) mg/l in HUU, p < 0.001, and 2.69 (1.90–4.29) mg/l vs 4.47 (2.96–5.71) mg/l in HUU, p = 0.001, respectively].

**Conclusion:**

Compared to HUU infants, HEU infants have IgG abnormalities mainly represented by low IgG2 levels, suggesting that despite maternal antiretroviral therapy, the mechanisms of IgG transplacental passage continue to be impaired in women living with HIV. HEU infants also showed a significantly lower level of specific anti-PCP IgG, possibly favouring a high vulnerability to *S. pneumoniae* infection at an age when protection is mostly depending on maternal IgG.

## Introduction

The prevalence of HIV infection is high in women of childbearing age in sub-Saharan Africa but, due to efficient health policies that expanded lifelong access to antiretroviral treatment in the past decade, the number of cases of pediatric HIV is constantly decreasing [1]. However, as a consequence, the number of infants born from women living with HIV who do not acquire the infection (HIV-exposed uninfected, HEU) has increased by more than half a million between 2018 and 2020 in the region [1]. HEU children are at an increased risk of morbidity, especially due to infectious causes, and have a 2–threefold higher mortality rate compared to their counterparts not exposed to HIV [2, 3]. This increased vulnerability is probably correlated to maternal HIV infection since, despite optimal antiretroviral therapy (ART), the functional immunological defects caused by HIV can not be completely reversed [4]. The maternal IgG transplacental transfer can have a crucial role in modulating the immune system of the infants and in protecting them from infections during the first 3–6 months of life, when infants have to rely only on maternal-derived immunoglobulins because of the inability to synthesize their own. Hypergammaglobulinemia, which is common in African mothers living with HIV [5, 6] has a significant impact on IgG transfer from the mother to the fetus [7]. Although the underlying mechanisms are not fully elucidated, it has been suggested that IgG levels over 15 g/l can cause the saturation of FcRN receptors, which mediate the IgG transplacental passage [8], and indeed, in clinical studies, maternal IgG levels over 15 g/l have been associated with abnormal IgG concentrations in HEU infants [9, 10]. Impairments of IgG transfer in HEU infants have been observed by some authors [5, 11–14], but others reported similar IgG levels in HIV-exposed and -unexposed infants [15, 16], or described a differential IgG passage depending on the characteristics of pathogen-specific antibodies [14]. The difficulty to obtain consistent results can be due to different causes, such as the timing of blood sampling (most of the mother/infant pairs studies have used cord blood whose composition mostly reflect fetal rather than neonatal condition [17]), or the lack of reference levels in healthy African infants. Moreover, IgG subclasses structures, glycosylation patterns, and polymorphisms are all factors potentially affecting transplacental passage [18, 19]. In a previous study, we reported the dynamic development of IgG in HEU infants from 1 to 24 months, finding abnormalities in concentrations and subclasses distribution. In particular, we observed persistently low IgG2 levels, but the lack of a proper control group and of long-term antiretroviral therapy of the mothers (the study was performed in the pre-Option B + era), prevented us to draw definite conclusions [20].

In the present study, we aimed to compare the total IgG levels and subclasses profile in two groups of 6-week old infants living in Malawi, one including HIV-exposed uninfected infants born from antiretroviral treated-mothers (HEU group) and one represented by infants born to HIV-negative mothers (HIV-unexposed, uninfected, or HUU group). Also, because of the potential impact on the development of pneumonia in these infants, we assessed the transplacental transfer of anti-*Streptococcus pneumoniae* total IgG and IgG2 subclass (generally considered associated with protection).

## Materials and methods

### Population characteristics

This study is part of a larger study (conducted between January 2019 and June 2021) aimed to assess the factors that determine maternal retention in programs for the prevention of vertical HIV transmission and to compare the health of HIV-exposed infants under Option B + with that of HIV-unexposed infants (including assessment of growth, evaluation of the immune response to vaccines, and of the incidence of infectious and non-infectious events up to 1 year of age). The main study enrolled 163 HEU and 72 HUU infants. For the present study we included all HIV-unexposed infants who had available samples collected at 6 weeks and an equal number of HIV-exposed infants (40 HUU and 40 HEU).

The mothers were enrolled at week 36 of pregnancy when demographic characteristics were recorded and clinical visits were scheduled. At delivery, and at monthly subsequent visits mother/child pairs data were collected including information about regular ART intake. The study was conducted within the structures of the DREAM (Drug Resource Enhancement against AIDS and Malnutrition) Program of the Community of S. Egidio, an Italian faith-based non-governmental organization. Three clinical sites were involved: the urban DREAM Center, in Mandala, Blantyre, and the semi-urban sites of Chileka and Machinjiri. Mother/child pairs of both groups were followed until 12 months from delivery.

Blood samples from 6-week old infants were collected from the plantar surface of the infants' heel and Dry Blood Spots (DBS) prepared by locally trained people. Using sterile lancets the drops of blood were absorbed onto each circle of Whatman 903 filter paper card. DBS were dried at room temperature for 4 h and then stored at − 20 °C, in individual ziplock bags containing a desiccant until shipment to the laboratories at the Istituto Superiore di Sanità in Rome, Italy, where the DBS were stored at  − 20 °C, until processing.

### Dried blood spot processing

Two spots from each card were punched out to obtain 20 micro-disks (diameter: 3.2 mm) using a pneumatic DBS Card Punch (Analytical Sales and Services Inc., Flanders, NJ). For elution, we used a methodology already described [21] with a modification, consisting of two steps of extraction. In the first step, the final 20 micro-disks were placed together into a low binding flat-bottom 24-well plate covered with a lid and incubated overnight at + 4 °C in 400 µl of elution buffer [Phosphate Buffered Saline (PBS 1 × Sigma Aldrich, Milan, Italy) + 0.05% Tween 20 (Sigma Aldrich, Milan, Aldrich) + 0.1% BSA (Sigma Aldrich, Milan, Italy) gently shaken with a bench-top shaker; after the first incubation, the soaked punches and elution buffer were transferred into the corresponding centrifuging system, consisting of a 15 mL centrifuge tube (Falcon Polypropylene Conical Tubes, Corning Science) that held a microtube (1.2 ml Corning Cluster Tubes, Salt Lake City, UT), and supported an uncapped 2.5 mL syringe barrel at the open end [22]. Samples were centrifuged at room temperature (RT) for 7 min 1,800 RPM. Eluate was transferred in 1.5 low-binding vials (Protein LoBind Tube, Eppendorf) and centrifuged (14,000 RPM, 15 min RT) to remove debris. In the second step, the remaining soaked punches were re-incubated with 200 µl of elution buffer overnight, to remove the remaining blood. The second eluate was processed as the first one, then added to the first elution product, to obtain a final volume of about 500 µl.

Based on previous reports [23], a 3.2 mm punch was considered to contain 3.275 µl of blood; considering a hematocrit value of 50% as acceptable for infants we calculated 1.6375 µl of plasma for each 3.2 mm punch. The final dilution was therefore of 1:18.3 or 32.75 µl (1.6375 µl × 20 spots) in 600 µl of elution buffer.

### Quantification of IgG and subclasses

An automatized nephelometry (BN ProSpec® System analyzer, Siemens Healthcare Diagnostics) was used for IgG determination. Total IgG and IgG subclass levels in DBS samples were analyzed using reagents from Siemens Healthcare Diagnostics. All subclasses determinations, but IgG2, were within the limit of detection. For IgG2 the limit of detection was 0.344 g/l and this value was used in the statistical analysis for samples with undetectable IgG2.

### Anti-pneumococcal IgG determination

Measurement of anti-pneumococcal IgG and anti-pneumococcal IgG2 were evaluated using commercial ELISA kits (VaccZyme anti-PCP IgG and VaccZyme anti-PCP IgG2 Enzyme immune Assay, Binding Site, Birmingham, UK) according to the manufacturer’s instructions. The detection limit was 3.3 mg/l for IgG and 1.1 mg/l for IgG2 and these values were used in the statistical analysis for samples with undetectable anti-PCP IgG or anti-PCP IgG2, respectively.

### Statistical analysis

For statistical analyses, the SPSS software, version 27 (IBM, Somers, NY, USA) was used. Results are presented as medians with interquartile range (IQR) and percentages. Differences between groups were evaluated using the *χ*^2^ test or Fisher’s exact test when appropriate for categorical variables, and by the Mann–Whitney *U* test for quantitative variables. Spearman’s correlation coefficient was used to evaluate correlations between quantitative variables. Differences were considered statistically significant when p < 0.05. IgG subclasses distribution was expressed as percentages of the different subclasses out of the total IgG.

## Results

### Women characteristics

Mothers’ characteristics are reported in Table 1. The women of the two groups did not differ for age; most of them were living in semirural areas, in similar socioeconomic conditions. All women living with HIV had received antiretroviral therapy (87.5% with tenofovir, lamivudine or emcitricitabine, and efavirenz) for a median of 19.5 months (IQR: 3.0–84.8) before enrollment. They all reported regular intake of ART at the delivery visit. All women with viral load data (n. 20) had HIV-RNA < 1,000 copies/ml, with the majority of women (n = 17) with HIV-RNA values below 40 copies/ml.

**Table 1:** Characteristics of enrolled Malawian women

	Mothers living with HIV	Mothers not living with HIV	P values
N	40	40	
Age (years)	30 (23.0–34.8)	28.5 (21.3–32.8)	0.415
Weight (kg)	61 (55.9–66.0)	67 (59.7–70.5)	0.086
Residence (n, %)			
Rural	10 (25)	16 (40)	0.184
Semirural	18 (45)	18 (45)	
Urban	12 (30)	6 (15)	
Education (n, %)			
None	3 (7.5)	3 (7.5)	0.477
Primary	16 (40)	20 (50)	
Secondary or above	21 (52.5)	17 (42.5)	
Available at home (n, %)			
Water	25 (62.5)	28 (70)	0.637
Electricity	16 (40)	16 (40)	1.000
ART duration at enrollment (months)	19.5 (3.0–84.8)		–
Therapy (n, %)			
TDF XTC EFV^1^	35, 87.5%	–	
Other	5, 12.5%		
Primigravidae (n,%)	8 (20.0)	7 (17.5)	0.500
Vaginal delivery (n, %)	33 (82.5)	32 (84.2)	0.541
Gestational age at delivery (weeks)	40.0 (38.3–42.0)	40.0 (38.0–42.3)	0.590
Pre-term delivery (n. %)	3 (7.5%)	3 (7.5%)	1.000

Values are expressed as medians with IQR or percentage

^1^Tenofovir, Lamivudine or Emtricitabine, Efavirenz

The forty women of the control group received the HIV test at a median of 3 months before enrollment (median: 94.0 days, IQR 46.5—132).

The gestational age at delivery was similar for the 2 groups of women (median 40 weeks), and vaginal delivery was the most common mode of delivery in both groups. Six pre-term births (before 37th week) occurred: 3 in the HEU group and 3 in the HUU group. All infants were exclusively breastfed in the first 6 months of life.

### Immunoglobulin G and isotypes in 6-week old infants

The analysis of IgG concentrations was performed from DBS of 6-week old infants. The results are reported in Table 2. HEU infants had significantly higher levels of total IgG compared to unexposed infants (6.87 vs. 5.79 g/l, p = 0.006), with a different subclasses distribution: a significant over-representation of IgG1 (5.48 g/l in HEU vs 4.67 g/l in HUU, p = 0.014) and a significantly lower level of IgG2 (0.378 vs. 0.485 g/l, p = 0.037). No differences were observed in IgG3 and IgG4 concentrations between groups. IgG1 levels were strongly correlated with the total IgG concentrations in both groups (HEU: r = 0.848, HUU r = 0.944, p < 0.001), while IgG2 levels were significantly correlated to the total IgG concentrations in HUU infants (r = 0.568, p < 0.001) but not in HEU infants (r = 0.095, p = 0.559).

**Table 2:** Total IgG, IgG subclasses and specific anti-PCP IgG in the 2 groups of 6 week-old infants

		HEU group (n = 40)	HUU group (n = 40)	P values
Total IgG	g/l	6.87 (6.13–7.90)	5.79 (5.06–7.14)	0.006
IgG1	g/l	5.48 (4.73–5.92)	4.67 (3.46–5.73)	0.014
IgG2	g/l	0.376 (0.344–0.523)	0.485 (0.374–0.781)	0.037
IgG3	g/l	0.162 (0.110–0.188)	0.164 (0.112–0.271)	0.225
IgG4	g/l	0.034 (0.200–0.775)	0.040 (0.026–0.081)	0.438
anti-PCP IgG	mg/l	8.9 (5.4–15.1)	16.2 (9.61–25.8)	0.001
anti PCP IgG2	mg/l	2.69 (1.80–4.29)	4.47 (2.96–5.71)	0.002

**Fig. 1:**
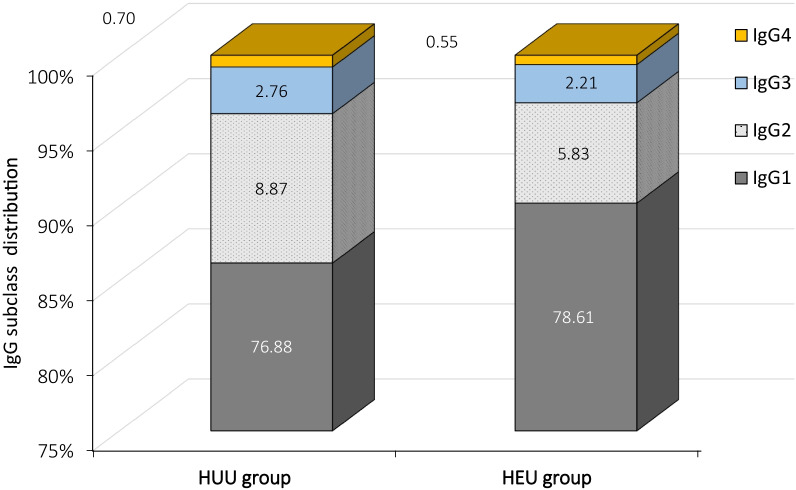
IgG subclasses distribution expressed as percentage of total IgG in the HEU and HUU groups

The IgG subclasses profiles were also different in the 2 groups of infants, with IgG2 representing 5.83% of total IgG in HEU and 8.87% in HUU infants (Fig. 1).

### Concentration of anti-pneumococcal IgG and IgG2 in the 6-week old infants

**Fig. 2:**
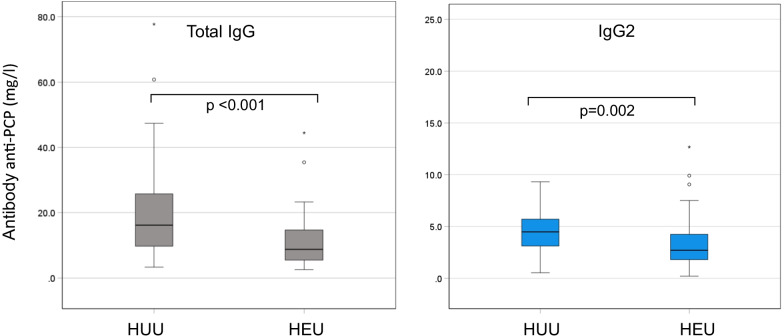
Anti-pneumococcal capsular polysaccharide IgG and IgG2 (anti-PCP IgG and anti-PCP IgG2) plasma concentrations in HEU and HUU infants. Values are expressed as medians with IQR

Six HEU (15%) and 3 HUU infants (7.5%) had anti-PCP IgG levels below the limit of detection (3.3 mg/l). The median anti-PCP IgG concentrations were significantly lower in HEU infants compared to HUU infants: 8.76 mg/l (IQR: 5.37–15.08) vs 16.17 mg/l (IQR: 9.61–25.83), respectively (p = 0.001) (Fig. 2).

Since IgG responses to bacterial capsular polysaccharide antigens are mostly restricted to IgG2, we also determined the concentrations of this isotype. Four HEU and 1 HUU had anti-PCP IgG2 levels below the limit of detection. Anti-PCP IgG2 median levels were significantly lower in HEU infants compared to HUU infants (2.70 vs. 4.47 mg/l, p = 0.002, Fig. 2). In both groups, a significant correlation was observed between the specific anti-PCP IgG and anti-PCP IgG2 concentrations (HEU: r = 0.666, p < 0.0001; HUU: r = 0.478, p = 0.002), and the ratio anti-PCP IgG2/anti-PCP IgG was similar in HEU and HUU infants (0.282 vs. 0.286, p = 0.624).

No correlation was found between anti-PCP IgG levels and the total IgG concentrations (HEU: r = 0.217, p = 0.187; HUU r = 0.167, p = 0.303), nor between anti-PCP IgG2 levels and the IgG2 concentrations (HEU: r = 0.067, p = 0.680; HUU r = 0.175, p = 0.280).

## Discussion

The primary objective of this study was to determine whether HEU infants born to ART-treated mothers had immunoglobulins (including specific anti-PCP IgG) levels and patterns, different from those of HUU infants. Our results suggest that 6-week old HEU infants still present immunoglobulins abnormalities in concentrations and distribution including low levels of specific anti-pneumococcal IgG and IgG2.

The main determinants of infant IgG profile, at 6 weeks of life, are the maternal IgG levels and distribution [14, 18]. In our study, we did not evaluate maternal samples, but it is well known that hypergammaglobulinemia and maternal immunological dysfunctions in the B compartment are common in African pregnant women living with HIV [5, 6, 10], and that the polyclonal B cell activation persist even after prolonged antiretroviral treatment [24, 25]. As we hypothesized in our previous work [6], maternal hypergammaglobulinemia could be responsible of the high levels of IgG in their infants. Indeed, in HEU we observed significantly higher levels of IgG and IgG1 compared to HUU infants, with a similar IgG1/IgG ratio in the two groups (HEU: 78.6%; HUU: 76.9%), indicating that the IgG1 distribution reflected that of total IgG. IgG1 is the most diffuse IgG subclass (about 60–70% in the general population [26]) and the preferential class in the transplacental transfer efficiency hierarchy, followed by IgG4, IgG3, and IgG2 [27]. In this study, we did not find significant differences in IgG3 and IgG4 concentrations between the two groups of infants, although the IgG3 proportion was slightly higher in the HUU group. Both subclasses have a not well-defined role in response to diseases, and abnormalities in their levels and distribution are often reported in combination with other IgG subclasses [28].

The significantly lower concentration of IgG2 in HEU infants (22% lower levels compared to HUU infants) and their lower representation out of the total IgG (-35% compared to the counterparts) is one of the relevant findings of this study; although mothers had received ART for a median of 19 months, the level of IgG2 was comparable to the one observed in HEU infants born to mothers treated with short-term ART [5, 29]. These findings suggest that the immunological functional defects due to HIV infection persist in women under continuous ART and interfere with the delicate balance of the maternal–fetal unit. The lack of correlation between total IgG and IgG2 in the HEU population (but not in HUU) is suggestive of a selective impairment of IgG2 passage through the placenta in mothers living with HIV.

The deficit of IgG2 has been associated with increased vulnerability to bacterial diseases [30, 31] since this subclass is responsible for the response against bacterial capsular antigens. We, therefore, extended our analyses to antigen-specific IgG against *S. pneumoniae*, which is a leading cause of lower respiratory infection among children younger than 5 years in low-income countries [32]. Vaccination against *S. pneumoniae* has been introduced in Malawi in 2011 and mothers of the study had not received this vaccine. HEU infants showed significantly lower levels of specific anti-PCP IgG compared to HUU infants, with a similarly low proportion of anti-PCP IgG2. To note that the anti-PCP IgG2/anti-PCP IgG ratio was similar in HEU and HUU infants (0.282 vs 0.286), suggesting that the transplacental passage of anti-pneumococcus IgG was globally lower in HEU infants, without a selective reduction of the specific anti-PCP IgG2.

Generally, higher total IgG correlates with higher pathogen-specific antibody levels [33]. In our study the lack of correlations between total IgG and anti-PCP IgG (and of IgG2 and anti-PCP-IgG2) was observed in both groups, suggesting a differential efficiency in the mechanism of IgG transplacental passage. Differences in the efficiency of transfer based on IgG subclasses structures and types of antigen-specific immunoglobulins has been previously reported [14, 27]. Contrasting results have been obtained in different studies on HEU infants: some reported reduction in transfer of specific antibodies against *H. influenzae*, diphtheria, pertussis, pneumococcus, measles, tetanus, and *Plasmodium falciparum* [10, 12, 16, 29, 34, 35], but others did not find any effect [15, 36]. The discrepancies between studies could be multifactorial, including methodology, different maternal ART coverage, different exposure to pathogens, the geographical areas, and the timing of infants analysis. One of the strengths of this study is that we determined IgG concentrations from the blood of 6-week old infants, which significantly differ from cord blood, the source used in most of the studies in this field [5, 7, 10, 12, 14–16, 34, 35]. Cord blood samples composition mostly reflects fetal rather than neonatal characteristics [17, 37, 38]; for instance, in cord blood generally, there are higher IgG levels than in mothers [39].

Our study has some limitations, including the limited numbers of infants in both groups and the lack of detailed information on the viro-immunological conditions in ART-treated mothers that could bias our results. However, considering the lifelong ART strategy, the available data on HIV-RNA for the study, and the high rate of treatment adherence observed in mothers it is highly likely that most of the mothers arrived at delivery with controlled HIV infection. Another important limit is that we could not correlate the levels of IgG anti-PCP found in infants with clinical data and the possible subsequent development of pneumonia. Lastly, the possibility to analyze maternal samples could have helped us in the interpretation of the results.

## Conclusions

Overall, our results support the notion that despite maternal ART administration under Option B + , the infants exposed to HIV still show immunoglobulin abnormalities in terms of concentrations and distribution compared to unexposed counterparts. The observed low levels of anti-PCP IgG may translate into higher vulnerability to *S. pneumoniae* infection in a population mostly depending on protective maternal IgG. These findings can form the basis for additional studies that are needed to elucidate the clinical impact that these abnormalities can have.

## Data Availability

The datasets used and analysed during the current study are available from the corresponding author on reasonable request.
